# Sexual dimorphism of liver metastasis by murine pancreatic neuroendocrine tumors is affected by expression of complement C5

**DOI:** 10.18632/oncotarget.8874

**Published:** 2016-04-20

**Authors:** Tanupriya Contractor, Shinta Kobayashi, Edaise da Silva, Richard Clausen, Chang Chan, Evan Vosburgh, Laura H. Tang, Arnold J. Levine, Chris R. Harris

**Affiliations:** ^1^ Raymond and Beverly Sackler Foundation Laboratory, New Brunswick NJ; ^2^ Institute for Advanced Study, Princeton NJ; ^3^ Department of Pathology, Memorial Sloan-Kettering Cancer Center, New York NY; ^4^ Rutgers University Cancer Institute of New Jersey and Department of Pediatrics, Robert Wood Johnson Medical School, New Brunswick NJ; ^5^ Department of Pediatrics, Robert Wood Johnson Medical School, New Brunswick NJ

**Keywords:** neuroendocrine tumors, pancreatic, metastasis, complement C5, sexual dimorphism

## Abstract

In a mouse model for neuroendocrine tumors of the pancreas (PanNETs), liver metastasis occurred at a higher frequency in males. Male mice also had higher serum and intratumoral levels of the innate immunity protein complement C5. In mice that lost the ability to express complement C5, there was a lower frequency of metastasis, and males no longer had a higher frequency of metastasis than females. Treatment with PMX53, a small molecule antagonist of C5aR1/CD88, the receptor for complement C5a, also reduced metastasis. Mice lacking a functional gene for complement C5 had smaller primary tumors, which were less invasive and lacked the CD68+ macrophages that have previously been associated with metastasis in this type of tumor. This is the first report of a gene that causes sexual dimorphism of metastasis in a mouse model. In the human disease, which also shows sexual dimorphism for metastasis, clinically advanced tumors expressed more complement C5 than less advanced tumors.

## INTRODUCTION

Sexual dimorphism has been reported for many types of human tumors. Often this is due to behavioral differences, such as higher tobacco use by males. But it is also becoming clear that hormonal/genetic differences account for sexual dimorphism of some tumors. Mouse models, where diet and environmental factors can be well-controlled, are particularly useful in demonstrating the sexual dimorphism of tumors. Human hepatocellular cancer shows sexual dimorphism due in part to higher tobacco use by males, but this disease is now thought to have a hormonal component as well because male mice were found to be more susceptible than female mice to the disease [1], due to higher IL-6 expression by males [2] but also due to the effect of FoxA1 and FoxA2 transcription factors on hormone-responsive promoters [3]. Nonmelanoma skin cancer also has a higher incidence in human males, which was replicated in a mouse model and subsequently linked to gender-specific expression of catalase [4].

The aforementioned diseases show sexually dimorphic rates of tumor incidence; for other tumor types, the rate of incidence is the same in males and females, but metastasis is sexually dimorphic. Two such diseases are melanoma [5] and neuroendocrine tumors, with males showing higher rates of metastasis in both diseases. In a large survey of patients with neuroendocrine tumors, males presented in the clinic with metastatic disease at a higher frequency (29%) than females (25%) (*p* < 0.01); males also had a lower rate of overall survival [6, 7].

Neuroendocrine tumors of the pancreas (PanNETs) are surprisingly common neoplasias. In an autopsy study of a general population of older adults, PanNETs were observed at a frequency of 10% [8]. Conversely this disease is rare in the clinic, although its frequency appears to be increasing [6, 7]. A key difference between clinical and autopsy frequency is the presence of metastasis. In autopsy studies, tumors are nearly always found only in the pancreas, whereas a high percentage of clinical patients present with metastatic liver disease. Patients with clinically diagnosed metastatic disease due to PanNETs only live an average of two years, whereas the outlook for pre-metastatic patients is much more favorable [7]. Therefore better understanding of how to prevent and treat liver metastasis is the key to improving outcomes for patients with PanNETs.

The RIP1-Tag2 (RT2) cancer mouse model has been the subject of a large number of studies, in part because it is one of the oldest cancer mouse models but also because tumorigenesis in RT2 mice is highly penetrant, reproducible, and reasonably recapitulates human pancreatic neuroendocrine tumorigenesis [9]. This transgenic mouse expresses the SV40 T-antigen under control of the rat insulin promoter, resulting in pancreatic beta cell tumors [10]. T antigen inactivates the Rb and p53 pathways, and inactivation of these pathways also occurs in human PanNETs, through amplification of negative regulators such as Cdk4 and Cdk6 [11], or WIP1 and MDM2 [12], respectively. Importantly, studies that showed a response by RT2 mice to sunitinib [13] and to rapamycin [14] predicted the success of subsequent clinical trials of patients with Pan-NETs [15, 16]. Antagonism of IGF1 receptor was unsuccessful as a treatment in a recent human trial [17]; similarly, antagonism of IGF1 receptor also failed to block tumor progression in RT2 mice, unless the mouse line was also knocked out for insulin receptor [18]. Therefore, the RT2 mouse is a rare example of a clinically-predictive and pharmacologically-validated animal model for a human disease.

In the RT2 model, mice can show metastatic liver disease by the age of 13–17 weeks. The frequency of metastatic disease in RT2 mice depends on the genetic background. Locally invasive tumors occur in more than half of 14 week old RT2 B6 mice, which derive from a C57Bl6 genetic background, but invasive tumors occur in less than 15% of 14 week old RT2 C3H mice, which derive from a C3H genetic background [19]. Metastasis in RT2 mice has been previously linked to the genes c-met, Alk, and CSF-1 [19–21]. These are probably just a small representation of a very large set of genes involved in metastasis, which is a complicated process that requires tumor cells to alter adhesiveness, change shape, become more motile, enter and then exit lymph or blood, and colonize a new organ [22–24].

We have studied metastasis of PanNETs in the RT2 mouse model but have used yet another genetic background, RT2 AB6F1. Importantly, as described below, metastasis was much more common in male RT2 AB6F1 mice than in females. RT2 AB6F1 mice are hybrids of the common inbred lab strains C57Bl6 (B6) and A inbred mouse lines, which have been previously been shown to differ in sensitivity to various pathogens [25–27], which was often due to the fact that A mice encode a frameshift mutation in the gene for the innate immunity protein complement C5 [28]. Complement C5 has also been linked to metastasis in several mouse studies [29–33]. Moreover, sexually dimorphic expression of complement C5 has been reported in C57Bl6 mice [34]. For these reasons we wondered if complement C5a contributed to the sexual dimorphism of liver metastasis in the RT2 AB6F1 mice.

## RESULTS

**Figure 1:**
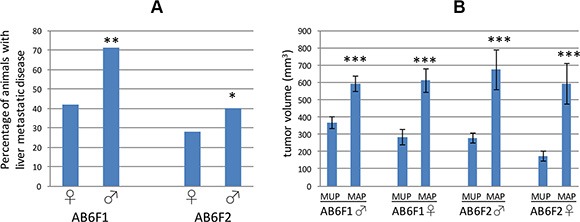
**(A)** The percentage of female (♀) or male (♂) RT2 mice demonstrating liver metastatic disease at 17 weeks of age is shown for two different genetic backgrounds, AB6F1 and AB6F2. For both genetic backgrounds, males were statistically more likely to develop liver metastasis. **indicates statistical significance with a *p* value below 0.01, and *indicates a *p* value below 0.05. *p* value was determined by Fisher's exact test. In total, we studied 190 RT2 AB6F1 males, 92 RT2 AB6F1 females, 155 RT2 AB6F2 males and 106 RT2 AB6F2 females. (**B**) Comparison of tumor volumes of metastasis-unassociated primaries (MUPs) found in mice without metastatic disease, and metastasis-associated primaries (MAPs) found in mice with liver metastasis. Mice were 17 weeks old, and data are separated by gender and genetic background. ***indicates a *p* value below 0.01 as determined by nonparametric Mann-Whitney analysis.

We mated male RT2 B6 mice, in which SV40 T antigen is expressed from the insulin promoter in a C57Bl/6 genetic background, to female mice from an inbred A/J background to generate a hybrid line designated RT2 AB6F1. In this nomenclature, “A” refers to the A/J mouse genetic background and “B6” is shorthand for the inbred C57Bl/6 mouse genetic background. Sibling RT2 AB6F1 animals were also intercrossed to establish an F2 generation, RT2 AB6F2, in which potential metastasis genes might be mapped due to the genetic differences between individual animals. The frequency of metastasis was higher in the RT2 AB6F1 line than in the F2 line (Figure 1A). Interestingly, in both RT2 AB6F1 and RT2 AB6F2 mouse lines, the frequency of metastasis was higher in males than in females (Figure 1A). In male and female mice from both genetic backgrounds, tumor size correlated with presence of metastatic disease (Figure 1B). The metastasis-associated primary tumors (MAPs) found in animals with liver metastatic disease were similar in size for both genders, and were significantly larger than the metastasis-unassociated primary tumors (MUPs) found in animals without liver metastatic disease.

**Figure 2:**
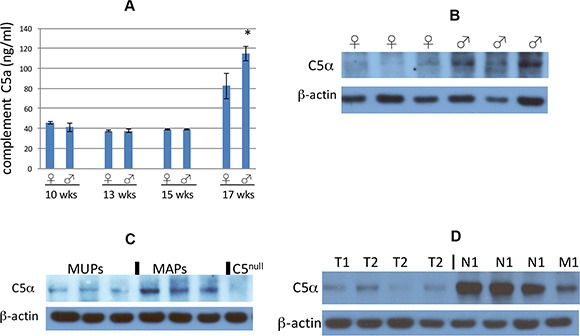
**(A)** Complement C5a in mouse sera samples was assayed by enzyme-linked immunosorbent assay, and plotted according to age and gender of the mice. The difference between male and female 17 week old mice was statistically significant (*p* < 0.05 by two tailed *t* test). The difference between 17 week old mice and younger mice was also statistically significant. Sera from a total of 34 mice were assayed. (**B**) Western blot of complement C5 alpha expression within primary tumors from six representative 17 week old mice, the first three of which are females and the next three of which are males. None of these six mice had liver metastatic disease at the time of euthanasia. (**C**) Western blot of complement C5 alpha expression within primary tumors from six representative 17 week old mice. The first three mice had no liver metastasis at the time of euthanasia, and the second three mice did show liver metastatic disease. An extract from a tumor isolated from RT2 AB6F2 mouse lacking a functional complement C5 gene is shown as a negative control. (**D**) Western blot analysis of eight representative primary human pancreatic neuroendocrine tumors for expression of complement C5 alpha. The first four tumors had a low pathological stage (T1 or T2, without local or distal metastasis) and the other four tumors had local metastatic disease (N1) or distal metastatic disease (M1).

As mentioned in the Introduction, the innate immunity protein complement C5 has previously been shown to affect metastasis of certain tumor types in mice and moreover to have gender-specific expression, so we wondered if this protein might account for the sexual dimorphism observed in RT2 AB6F1 mice. Complement C5 is a circulating protein, so we measured serum levels of complement C5a in RT2 AB6F1 mice. Interestingly in younger RT2 AB6F1 mice, serum levels of complement C5a did not differ between males and females, but expression grew significantly in 17 week animals, and 17 week old males produced more complement C5a than females (Figure 2A).

Primary tumors from RT2 AB6F1 mice were also assayed for complement C5 protein. Complement C5 is composed of two polypeptide chains, complement C5 beta and complement C5 alpha; complement C5 alpha is subsequently processed into two additional polypeptides, complement C5 alpha' and C5a [35]. Complement C5 beta and C5 alpha' form a complex called complement C5b, which together with other complement subunits form the membrane attack complex that lyses invading bacterial pathogens. Complement C5a is a powerful chemokine but is notoriously short-lived. Indeed, complement C5a could not be detected in the tumors by Western blot. But we were able to observe the precursor, complement C5 alpha. Male tumors expressed more complement C5 alpha than female tumors (Figure 2B). The expression of complement C5 alpha also correlated with presence of liver metastatic disease, as there was more complement C5 alpha in MAPs than in MUPs (Figure 2C).

We obtained 37 human pancreatic neuroendocrine tumors from the Cooperative Human Tissue Network. Twenty-two of the samples were from male patients and 15 were from females; 13 of the samples were from an early pathological stage of the disease (T1 or T2) and 24 were more advanced, (T3, N1 or M1). All were Caucasian except for two African American patients. Ages ranged from 25–74, but 51% of the patients were in a narrow age range between 49 to 61. In the human tumors, the short-lived complement C5a species could also not be detected by Western blot, but complement C5 alpha was highly expressed within some of the primary tumors. Notably the tumors with highest expression of complement C5 alpha were also the tumors with the highest pathological stage: whereas tumors staged at T1 or T2 and lacking lymph node or distal metastasis expressed very little complement C5 alpha, tumors with pathological staging of T3, T4, N1 or M1 had much higher expression (Figure 2D). This result suggests that complement C5 could affect the progression of neuroendocrine tumors in humans, as well as in mice.

**Figure 3:**
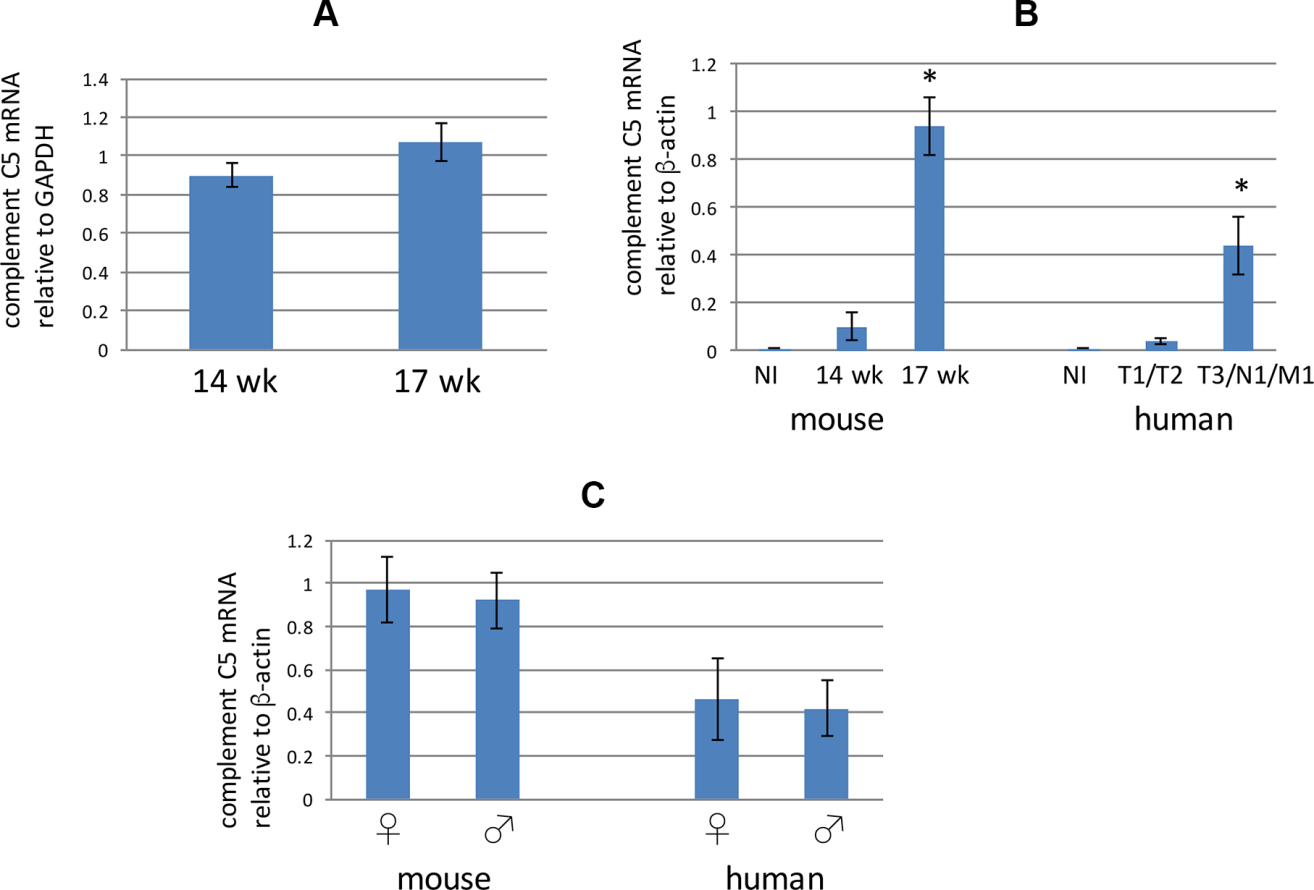
**(A)** Complement C5 mRNA from liver extracts prepared from 14 week old or 17 week old RT2 AB6F1 mice was assayed using quantitative RTPCR. Differences were not statistically significant. Livers from 39 different mice were used for this experiment. (**B**) Analysis of complement C5 mRNA in normal beta islets (NI) isolated from mice, or from tumors isolated from 14 week mice, or from more advanced tumors from 17 week mice. Also, complement C5 mRNA was measured in normal beta islets (NI) isolated from human donors, or from less advanced human PanNETs (T1 or T2 pathological stage, without local or distal metastasis) or from more advanced human PanNETs (local or distal metastasis, and/or pathological stage T3). *indicates *p* < 0.05 as determined by two-tailed *t*-test. One way ANOVA was also applied for the two data sets, and revealed a *p* value below 0.05 for both mouse and human samples. A total of 20 mouse tumors were used for this experiment, as well as normal islets from three animals. For human samples, 37 tumors were used, as well as normal islets from two donors. (**C**) Comparison of complement C5 mRNA in primary tumors from 17 week old male and female mice, and from clinically advanced human tumors. Differences were not statistically significant. Of the 58 mice sampled, 24 were isolated from males. Of the 24 human tumors used for this study, 13 were isolated from males.

Because liver is a major source of complement C5 [36], we assayed transcription of complement C5 within livers of male 14 week old and 17 week old RT2 AB6F1 mice. There did not appear to be a difference in complement C5 mRNA in livers from these different animals (Figure 3A), suggesting that liver may not be the source of the higher complement C5a found in sera from 17 week old mice (Figure 2A). However we did find an increase in transcription of the complement C5 gene in the primary tumors from 17 week old mice, as compared to the primary tumors from 14 week old mice (Figure 3B). Therefore, a portion of the increase in complement C5 species observed both in serum and within tumors of older mice may derive from production of these proteins within the tumors themselves. Moreover, a similar result was found for the human samples, where tumors at higher pathological stages expressed more mRNA for complement C5 than less advanced tumors (Figure 3B). Interestingly, transcription of complement C5 mRNA was equally high in advanced female and male tumors from both mice and humans (Figure 3C).

**Figure 4:**
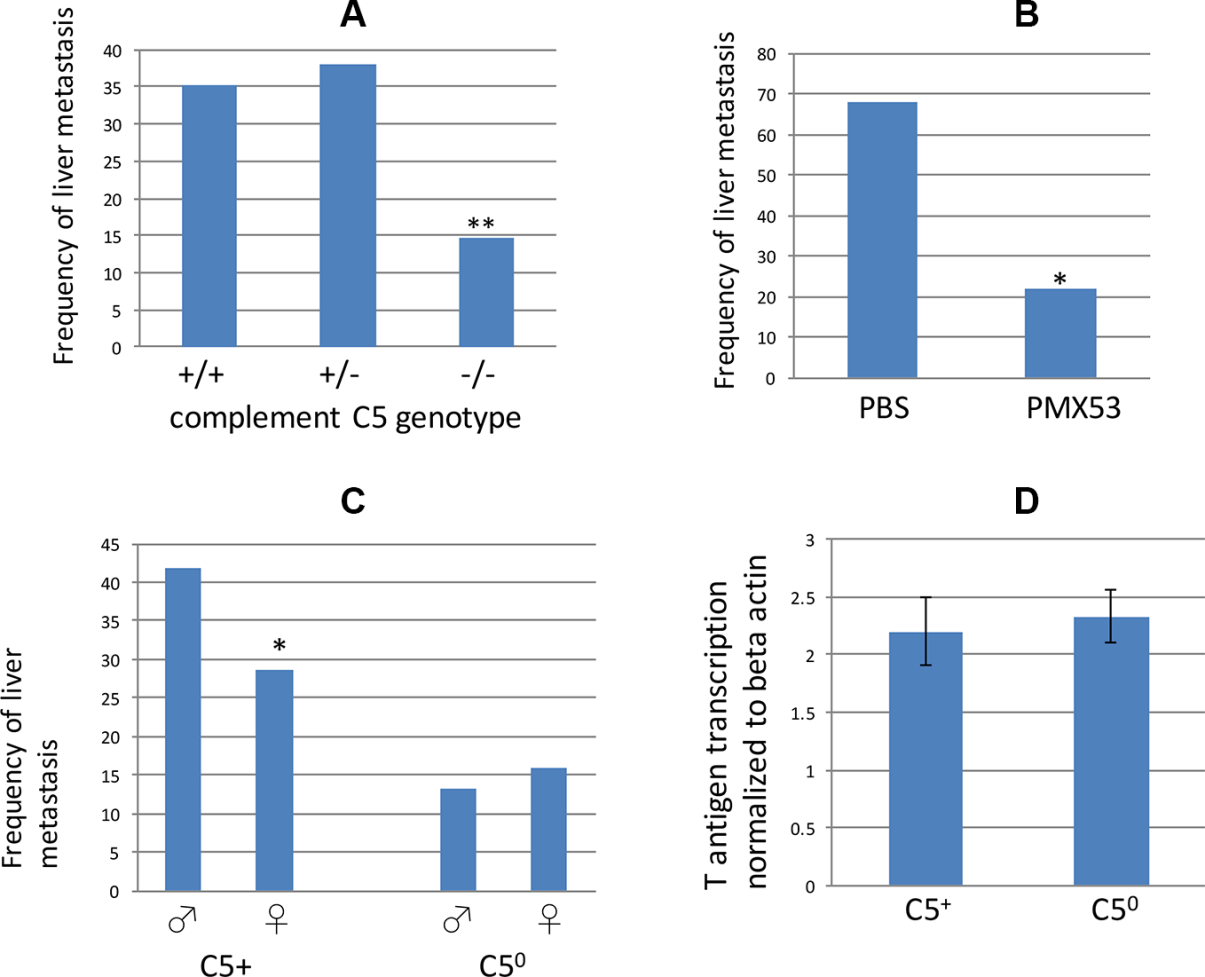
**(A)** Frequency of liver metastatic disease in RT2 AB6F2 mice, sorted by complement C5 genotype. −/− indicates animals that encoded two copies of a functionless complement C5 gene; +/+ animals had two functional copies of complement C5, and +/− animals were heterozygotes. A total of 265 mice (142 males and 123 females) were analyzed. **indicates *p* < 0.01; statistical significance was determined by Fisher's exact test. (**B**) A total of 34 male RT2 AB6F1 mice were treated for six weeks either with phosphate buffered saline or with PMX53, which inhibits the C5a receptor C5aR1/CD88. Treatment began at the age of 10 weeks and animals were euthanized and scored for presence of liver metastatic disease when they reached 17 weeks. Statistical significance was determined by Fisher's exact test. (**C**) Frequency of metastatic liver disease in 142 male or 123 female RT2 AB6F2 mice that have either lost both functional alleles of the complement C5 gene (C5^0^) or else have at least one functional complement C5 gene (C5^+^). *indicates *p* < 0.05 as determined by two-tailed *t*-test. The gender difference within C5^0^ mice was not statistically significant. (**D**) Transcription of SV40 T-antigen did not differ statistically within tumors of C5^+^ and C5^0^ mice.

As mentioned in the Introduction, A/J mice encode a mutant allele of complement C5, and therefore all RT2 AB6F1 hybrid mice inherit one functional copy of the complement C5 gene from their B6 fathers and one mutant copy from their A/J mothers. But in the F2 generation, mice can inherit zero, one, or two mutant copies of complement C5. Therefore within the RT2 AB6F2 mice, we looked for an association between ability to encode functional complement C5 and the presence of liver metastasis. As shown in Figure 4A, RT2 AB6F2 animals encoding two nonfunctional alleles of complement C5 had a significantly lower frequency of liver metastatic disease than animals encoding one or more functional copies of the gene. As measured by mRNA levels, expression of the SV40 T antigen oncogene was similar in RT2 AB6F2 mice regardless of whether they had a functional gene for complement C5 (Figure 4D).

To rule out the possibility that metastasis is caused by a gene near the complement C5 locus and not by the complement C5 gene itself, male RT2 AB6F1 mice were treated with PMX53, a small molecule inhibitor of C5aR1/CD88, the receptor for complement C5a [37]. Mice injected with PMX53 three times a week beginning at 10 weeks of age were less likely to develop liver metastatic disease than mice treated with vehicle (Figure 4B). Thus both genetic and pharmacological evidence link complement C5 to liver metastatic disease in RT2 mice.

Importantly, RT2 AB6F2 mice lacking a functional gene for complement C5 no longer showed a gender bias toward metastasis (Figure 4C). Complement C5 expression therefore not only promoted metastasis of tumors in RT2 mice, but was necessary for the higher frequency of metastasis observed in males. However it is also important to note that females as well as males benefited from loss of complement C5, although to a lesser degree.

**Figure 5:**
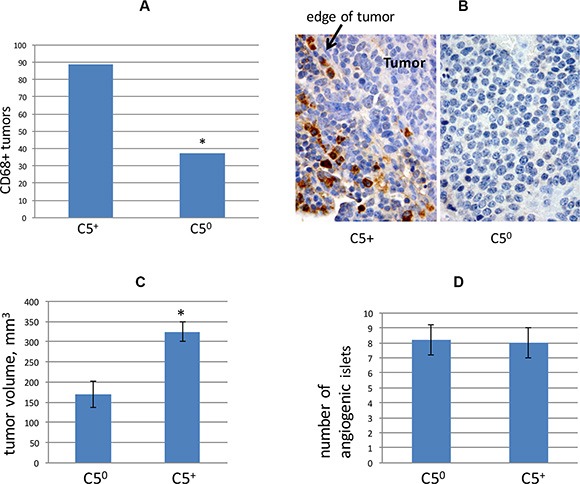
**(A)** Primary tumors from RT2 AB6F2 mice, with or without functional complement C5, were scored for presence or absence of CD68+ macrophages. *indicates *p* < 0.05 as determined by Fisher's exact test. Tumor samples from 17 mice were used for this analysis. (**B**) Representative immunohistochemistry of CD68 in C5null (C5^0^) and C5-encoding RT2 AB6F2 mice. When present, CD68-positive cells localized to the edge of tumors. (**C**) Tumor volumes were determined for RT2 AB6F2 mice with and without functional complement C5. Statistical significance was determined by non-parametric Mann-Whitney analysis, which revealed *p* < 0.05. A total of 182 mice were analyzed. (**D**) Angiogenic islets were counted in a set of 10 week-old RT2 AB6F2 mice encoding at least one functional complement C5 gene (C5+), or no functional complement C5 gene (C5^0^). Four mice of each genotype were analyzed.

Complement C5 is a chemoattactant for macrophages [38], and infiltration of neuroendocrine tumors by CD68+ macrophages has previously been reported to correlate with metastasis both in RT2 mice and in humans with pancreatic neuroendocrine tumors [21]. We prepared paraffin sections from tumors of C5 null and C5-encoding RT2 AB6F2 mice. Tumors from C5 null mice were less likely to stain for CD68-positive macrophages than tumors from C5-encoding RT2 AB6F2 mice (Figure 5A). Interestingly, in tumors from RT2 AB6F2 mice, the CD68+ cells appeared to be immediately adjacent to the tumors, and not directly infiltrating them (Figure 5B).

We also note that primary tumors were smaller in RT2 AB6F2 mice that did not encode a functional complement C5 gene (Figure 5C). Both complement C5 null and C5-encoding mice produced equal numbers of angiogenic islets at age of 10 weeks (Figure 5D). The appearance of angiogenic islets in 10 week old mice is a singular feature of the RT2 mouse model and reflects the early angiogenic events that occur during tumorigenesis [39]; the data in Figure 5D essentially rule out early angiogenesis as a cause of increased metastasis in complement C5-expressing mice.

**Figure 6:**
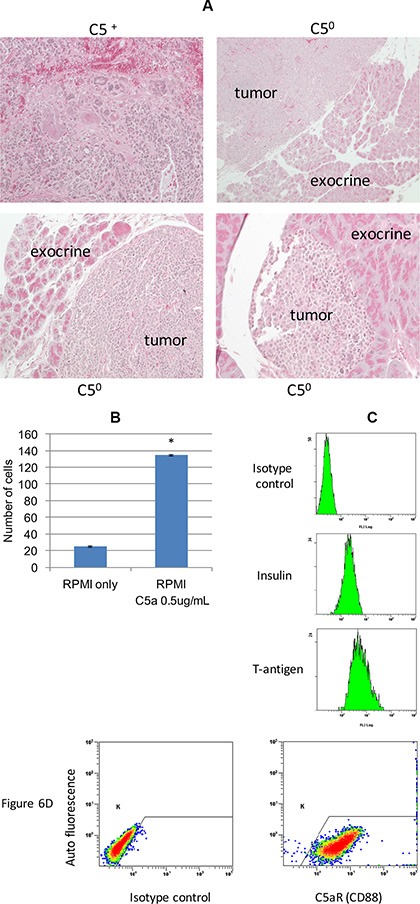
**(A)** Tumor samples from three C5 null (C5^0^) RT2 AB6F2 mice, each of which showed no invasion into the adjacent exocrine tissue. In comparison, a highly invasive tumor from a C5-encoding RT2 AB6F2 mouse is shown. (**B**) RT2 AB6F1 tumor cells were isolated for *in vitro* experiments, as described in materials and methods. The ability of the isolated tumor cells to invade matrigel was affected by the addition of recombinant mouse complement C5a. (**C**) *In vitro* expression of insulin and SV40 T-antigen proteins by isolated RT2 AB6F1 tumor cells, as determined by flow cytometry. (**D**) Expression of the receptor for complement C5a by isolated RT2 AB6F1 tumor cells, as determined by flow cytometry.

In 17 week old RT2 AB6F2 mice with a normal gene for complement C5, tumors were very large and often highly invasive, such as the tumor shown in the upper left panel in Figure 6A. Conversely, several tumors from 17 week old mice lacking complement C5 were not invasive (Figure 6A). To further investigate the effect of complement C5a on pancreatic neuroendocrine tumor invasiveness, we prepared cells from a primary tumor isolated from an RT2 AB6F1 mouse showing liver metastatic disease. After a month of growth in media designed to restrict growth of fibroblasts, we observed no fibroblasts or immune cells within the culture (data not shown) and the cells were homogeneous with respect to high expression of SV40 T antigen and insulin (Figure 6C), as well as the receptor for complement C5, C5aR1/CD88 (Figure 6D). The cultured tumor cells invaded matrigel *in vitro*, a property that was strongly stimulated by addition of recombinant mouse complement C5a (Figure 6B). This experiment suggests that complement C5a can increase the invasiveness of neuroendocrine tumor cells directly, in addition to effects on attraction of macrophages.

## DISCUSSION

We describe a mouse, RT2 AB6F1, which proved to be a useful model for studies on metastasis of pancreatic neuroendocrine tumors. There is a higher frequency of liver metastasis in RT2 AB6F1 mice than in previously described models of pancreatic neuroendocrine tumorigenesis [19]. Higher frequency of metastasis may facilitate the testing of often expensive compounds for anti-metastatic activity, because fewer mice might be required to see statistically significant effects. The sexual dimorphism of metastasis within RT2 AB6F1 mice mimics the sexual dimorphism of metastasis previously reported for the human disease [7], allowing the study of this property in an animal model. The fact that male bias of metastatic disease associated with neuroendocrine tumors can now be modeled in laboratory population of mice does not prove, but is highly suggestive that the human disease stems from hormonal/genetic differences, rather than gender-specific behavioral differences. We are not aware of any previous reports showing gender effects on metastasis in mice for any tumor type.

We have shown that the innate immunity protein, complement C5, promotes metastasis in RT2 AB6F1 mice, and is required for the sexual dimorphism. This was demonstrated with a complement C5aR1/CD88 antagonist, as well as by measuring linkage of metastasis to a functional gene for complement C5 in a genetic cross. We found two possible mechanisms by which complement C5 may increase the frequency of metastasis. Presence of CD68+ macrophages within tumors have previously been linked to metastasis of mouse and human pancreatic neuroendocrine tumors [21], and we have shown that tumors from C5-encoding animals display CD68+ macrophages along the tumor edges. Macrophages on the edges of other types of tumors are thought to contribute to tumor invasiveness and metastasis [40]. Complement C5a also increased the invasiveness of tumor cells grown in the absence of immune cells (Figure 6), and tumors were less invasive in animals that did not encode complement C5. Thus metastasis could be due to invasiveness, attraction of macrophages, or both. It should also be pointed out that complement C5 may produce M2 (immunosuppressive) macrophages [41], which have been linked to progression of tumors in RT2 mice [42], and this possibility has not yet been tested in these tumors. Finally we recently found that tumor cell dedifferentiation is important for metastasis in RT2 mice, and there may be some effect by complement C5 on this process (Kobayashi, Contractor and Harris, unpublished data). Because of the many potential properties affected by complement C5, unraveling the activity most important for metastasis as well as the specific molecular mechanism by which this activity occurs will require additional study.

As mentioned above, the gender bias of pancreatic neuroendocrine tumor metastasis was lost in mice null for the complement C5 gene (Figure 4C). Serum levels of complement C5a were higher in RT2 AB6F1 male mice at 17 weeks of age (Figure 2A), and more complement C5 alpha protein was detected within tumors from male mice (Figure 2B). Complement C5 alpha expression also increased in mouse tumors isolated from animals with liver metastatic disease, relative to animals without liver disease (Figure 2C). Our data also suggest that the increase in complement C5 protein species within primary tumors and serum derives at least in part from increased transcription of the complement C5 gene within tumors themselves (Figure 3B), whereas liver transcription did not appear to change (Figure 3A). But we still do not fully understand the precise mechanism by which a gender difference in complements C5a and C5 alpha expression occurs, as transcription of complement C5 mRNA was similar in tumors from both males and females (Figure 3C). Several mechanisms could be responsible, including increased proteolytic activation of complement C5 in males, increased post-translational stability of complement C5 in males, or higher transcription of complement C5 in males from a tissue other than tumor or liver.

Complement C5 expression also correlated with metastasis in the human disease. Clinically advanced human tumors expressed more complement C5 alpha protein than did less advanced tumors, and also transcribed more complement C5 mRNA. Unlike the mouse model, where complement C5 alpha expression was clearly higher in male tumors, we did not observe a significant gender difference between complement C5 alpha protein expression in human tumors by Western blot (data not shown). Perhaps complement C5 only accounts for sexually dimorphic metastasis of PanNETs in mice and not in humans. But another explanation is possible. Human populations are much more genetically diverse than inbred mice, and it took many thousands of patients in order to observe the sexual dimorphism of metastasis in the human disease [7]. If a large enough set of human pancreatic neuroendocrine tumors could be tested for differences in intratumoral expression of complement C5 alpha, it is quite possible that the genetic diversity problem could be overcome and that gender differences might then be observed. Unfortunately pancreatic neuroendocrine tumor specimens were mostly available only as paraffin sections, and complement C5 antisera were not satisfactory for staining paraffin sections, so it was not possible to run a larger study.

Complement C5 is a central player in innate immunity, a process that prevents many bacterial, fungal and viral infections [38]. Ironically, many of the properties of complement C5 that aid in fighting infection within otherwise healthy individuals might be expected to increase metastasis in individuals with tumors. For instance, metastasis requires tissue remodeling and local invasiveness as well as intravasation of tumors cells into blood and lymph, and extravasation to sites of metastasis; similarly in response to infections complement C5a helps to remodel tissue to increase invasiveness of immune cells, and increases extravasation and intravasation of immune cells both into the infected tissue and into blood and lymph nodes. Tumor associated macrophages often increase metastasis, including in human PanNETs [21, 42]; similarly complement C5a attracts macrophages to sites of infection. In previous studies complement C5 affected tumor progression at the level of the primary tumor, by increasing invasiveness [29] or angiogenesis [31]. But complement C5 also aided the more distal steps of the metastasis process, by allowing extravasation of fibrosarcoma cells into the lung [30], or by altering the immune cell environment of the metastatic target organ [32].

Pharmacological targeting of complement C5 might benefit patients at risk for developing metastasis. As mentioned above, the small molecule PMX53 blocked liver metastasis in RT2 AB6F1 mice. PMX53 is not in clinical development, but an antibody that inactivates complement C5 has been approved for use against two rare diseases, paroxysmal nocturnal hemoglobinuria and atypical hemolytic uremic syndrome [43]. Given its central role in innate immunity, targeting of complement C5 might also lead to infectious disease, so it will be important to determine if a risk of infectious disease might be outweighed by the potential benefit of preventing metastasis in certain patients.

## MATERIALS AND METHODS

### Human and mouse experiments

Human pancreatic neuroendocrine tumors were provided by the Cooperative Human Tissue Network (CHTN). Human experiments were approved by the Institutional Review Boards of CHTN. Normal islets from human donors were purchased from Beta-Pro LLC. Mouse experiments were approved by the Institutional Animal Care and Use Committee of Rutgers University. RT2 B6 mice were obtained from the National Cancer Institute (Frederick MD). A/J mice were purchased from Jackson Laboratories (Bar Harbor ME). Mice were maintained on a 12 hours light/dark cycle. Mice were switched to a high sucrose chow (TD.86489; Harlan Laboratories, Madison WI) at 12 weeks of age. At 17 weeks of age, mice were euthanized with carbon dioxide, and autopsied. Primary tumor volume was measured by caliper, and sections of primary tumors were quickly frozen in liquid nitrogen prior to protein and RNA analysis, or fixed in formalin for tissue staining. Livers were visually examined for signs of metastasis, and in certain cases stained paraffin sections were analyzed by a pathologist (L.H.T.) to confirm presence or absence of metastasis. Angiogenic islets were quantitated from euthanized 10 week old mice, as previously described [44]; islets from nontumorigenic mice were also isolated in a similar fashion, but were then handpicked and used for RNA extraction. Blood was collected from mice euthanized at 10, 13, 15 or 17 weeks of age, and used to prepare serum for measurement of complement C5a. The small molecule inhibitor of complement C5aR1, PMX53, was a generous gift from J. Lambris (University of Pennsylvania). PMX53 was dissolved in phosphate-buffered saline, and injected subcutaneously three times a week at a concentration of 0.55 mg/kg. PMX53 treatments began when mice reached 10 weeks of age, and continued until mice were 17 weeks. A control population of mice was mock injected with PBS three times a week.

### Genotyping

To assay complement C5 genotype, mouse tail DNA was assayed for mouse SNP rs27169032 using the following oligonucleotides: outside primers 5′CAGCCAAGGCTTTAGGTACAGT and 5′ACCTTAAT TTCGACCAGCAAGTGAA, and SNP-specific primers 5′VIC-ATGGTAGCCAATCCTAATG-NFQ and 5′FAM-TGGTAGCCAATTCTAATG-NFQ. The SV40 T antigen transgene of RT2 is tightly linked to the Rag1 gene on mouse chromosome 2 [45], and complement C5 is also on chromosome 2, 65 megabases away from the Rag1 gene. Of the RT2 AB6F2 progeny, 13% encoded two copies of the C5 null allele, instead of the 25% frequency that would be expected if the genes were unlinked

### Analysis of RNA and protein

Protein and RNA were assayed for individual tumors; tumor samples were never pooled. Taqman QPCR assays were purchased from Life Technologies, and RNA was prepared from snap-frozen tumors using an RNAeasy kit (Qiagen). Protein from snap-frozen human or mouse tumors was prepared in ice-cold RIPA buffer containing protease inhibitor cocktail 2 (Sigma) and phos-stop (Roche). After addition of RIPA, tumor tissue was homogenized for three minutes, on ice, using a Polytron PT1200E tissue grinder, then sonicated at 30% power for 30 seconds using a 50 Sonic Dismembrator (Fisher Scientific). Protein samples were then centrifuged at 12,000 rpm for 20 minutes, and insoluble pellets were discarded. Proteins were detected after Western blotting using antibodies purchased from R&D Systems (human complement C5 alpha), Sigma Chemicals (human beta actin, mouse beta actin), or Santa Cruz Biotech (mouse complement C5 alpha). Immunohistochemistry of CD68 was performed as previously described [21]. CD68 antisera were purchased from Serotec. An enzyme-linked immunosorbent assay for mouse complement C5a was purchased from Raybiotech.

### Statistical analysis

For RNA expression, measured values for a large number of individual tumors were used to generate a population, and differences between these populations were evaluated by two-tailed *t test*. For tumor volumes, the populations were evaluated by nonparametric Mann-Whitney analysis.

### *In vitro* analysis

Tumor cells were cultured from a primary pancreatic tumor isolated from a 17 week old RT2 AB6F1 male mouse; this mouse showed liver metastasis at the time of euthanasia. To deter growth of normal fibroblasts, the tumor cells were grown in a stem cell medium for several weeks, consisting of Dulbecco's modified Eagle's medium/F12 medium (Life Technologies) supplemented with N-2 supplement (Life Technologies), 20 ng/ml human epidermal growth factor (Life Technologies), 10 ng/ml human basic fibroblast growth factor (Sigma-Aldrich), 4 μg/ml heparin (Sigma-Aldrich), 4 mg/ml bovine serum albumin (Life Technologies), 20 μg/ml human insulin, zinc solution (Life Technologies), and 2.9 mg/ml glucose (Sigma-Aldrich) [46]. After several passages, the cells were switched to RPMI media with 10% fetal bovine serum. SV40 T-antigen antibody (Abcam), insulin antibody (Cell signaling) and CD88 antibody (Biolegend) were used for flow cytometry analysis. Invasiveness was tested using a matrigel invasion chamber (Corning) with or without addition of recombinant mouse C5a protein (R&D Systems). The invasion assay was repeated three times.
